# Equitable aging in health framework: a multi-systems and multilevel approach to health challenges and supports for transgender older adults

**DOI:** 10.1093/geroni/igaf103

**Published:** 2025-09-23

**Authors:** Angela K Perone, Leyi Zhou, Tré Coldon, Michael Solorio, Alec Paget, Ashlee Osborne

**Affiliations:** School of Social Welfare, University of California, Berkeley, California, United States; School of Social Welfare, University of California, Berkeley, California, United States; School of Social Welfare, University of California, Berkeley, California, United States; School of Social Welfare, University of California, Berkeley, California, United States; School of Social Welfare, University of California, Berkeley, California, United States; School of Social Welfare, University of California, Berkeley, California, United States

**Keywords:** Immigration, Intersectionality, Race, HIV, Housing, Employment

## Abstract

**Background and Objectives:**

While research on transgender older adults and health is growing, gaps remain about transgender older adults of color, immigrants, and other groups experiencing multiple forms of marginalization who are shaped by concurring experiences of oppression across the life course. This article aims to address these gaps by examining health challenges and supports among transgender older adults—many of whom are racially minoritized and immigrants—through an Equitable Aging in Health framework.

**Research Design and Methods:**

This community-driven study incorporates qualitative data from 37 transgender older adults from a larger study of 23 focus groups with 208 lesbian, gay, bisexual, transgender, queer, intersex, and asexual (LGBTQIA+) older adults in California to examine challenges, thriving and surviving strategies, and recommendations regarding health, housing, social services, and caregiving. Data foreground experiences of transgender older adults who are racially underrepresented, immigrants, and have low incomes.

**Results:**

Transgender older adults identified challenges related to healthcare access, housing, employment, economics, and violence that often intersected with disability and aging. Transgender older Latina immigrants experienced elevated challenges related to language barriers, immigration status, and discrimination. Supports included community connections, financial and legal assistance, educational workshops, and homesharing programs. Healthcare access, health experiences, and overall well-being were intricately tied to challenges and supports in housing, social services, healthcare systems, and employment that existed at micro, mezzo, and macro levels.

**Discussion and Implications:**

The Equitable Aging in Health framework helps illuminate how challenges and supports described by transgender older adults, including immigrants and older adults who are racially minoritized, can shape health-related experiences for transgender older adults. Policies, services, and programs targeting transgender older adults, thus, would benefit from a multi-level, multi-systems approach.

Innovation and Translational Significance: This community-engaged and theory-driven qualitative study builds on emerging research about health disparities among transgender older adults with a focus on low-income and racially minoritized older adults and immigrants—an area with sparse research. In-depth analysis and application of the Equitable Aging in Health conceptual framework demonstrated how challenges and supports in healthcare, housing, social services, and employment were interconnected and shaped experiences at the individual, organizational, and systems levels. This research underscores the importance of addressing these interconnected systems and levels to produce culturally responsive services, programs, and policies to better serve transgender older adults.

## Background and objectives

While research on LGBTQIA+ older adults is growing, a gap remains for transgender older adults (Asti et al., 2024; Henry et al., 2020), particularly transgender older adults who are racially underrepresented, immigrants, and who have low incomes (and often experiencing intersections of these social locations). Existing research underscores that transgender older adults face elevated health disparities and concerns, including higher rates of suicidal ideation (Fabbre & Siverskog, 2019; Grant et al., 2011), HIV and other sexually transmitted infections (STIs) (Coleman et al., 2022; Stutterheim et al., 2021) and cardiovascular disease (Asti et al., 2024) than the general population, which may be attributed to higher rates of smoking (Balcerek et al., 2021), hormone therapy (Getahun et al., 2018; Gooren & T’Sjoen, 2018; Nota et al., 2019), HIV (Feinstein, 2021), and other health disparities (Ceolin et al., 2024; Fredriksen-Goldsen et al., 2014a). Transgender older adults also experience worse health, disability, and perceived stress and depressive symptoms compared to cisgender (non-transgender) lesbian, gay, and bisexual older adults (Fredriksen-Goldsen et al., 2014a; Pharr, 2021). While many of these studies were conducted in the United States, research on transgender aging in Canada (Kiran et al., 2019) and the United Kingdom (Benbow et al., 2021) has produced similar findings.

Additional barriers to care include access and delayed care (Beehuspoteea & Badrakalimuthu, 2021), limited knowledge and training among healthcare professionals to provide culturally responsive care (Beehuspoteea & Badrakalimuthu, 2021; Benbow et al., 2021; Finkenauer et al., 2012; Willis et al., 2020), limited access to specialist services (Witten, 2014b), and discrimination (Benbow & Kingston, 2022) through denial of care (Walker et al., 2023), policy (Perone, 2020), and verbal or physical abuse (Finkenauer et al., 2012). Transgender older adults have also expressed concerns about discrimination and quality care in residential long-term care, including nursing homes (or care homes) in Sweden (Siverskog, 2015), Canada (Kortes-Miller et al., 2018; Pang et al., 2019) and the United States (Justice in Aging, 2015; Knochel & Flunker, 2021, Witten, 2014a) and prompted an international group of scholars to propose standards of care for serving transgender older adults in institutional environments (Coleman et al., 2022). These barriers can also exacerbate mistrust of healthcare providers and services among transgender older adults (Willis et al., 2020; Witten, 2014b), including concerns about being “outed” by healthcare providers or their staff (Dhillon et al., 2020; Willis et al., 2020). Barriers to accessing care are exacerbated for transgender older adults experiencing multiple forms of marginalization (e.g., transgender older adults of color) (Walker et al., 2023).

Despite increasing evidence of health disparities and discrimination, many transgender older adults have a long history of resistance to some of these structural barriers (Li et al., 2023), resilience (Fredriksen-Goldsen et al., 2024; Sloan & Benson, 2022; Witten, 2014b), and strong social networks and community supports (Fabbre & Gaveras, 2020; Toze et al., 2023; Witten, 2014b). Li et al. (2023) write that trans older adults develop what they call authenticated social capital by re-negotiating social barriers and creating alternative and affirming social networks and supports. Supportive networks may help buffer some of these challenges (Fabbre & Gaveras, 2020) and provide a space for joy (Adan et al., 2021; Fabbre, 2015).

While a body of evidence on transgender older adults and health is growing, gaps remain about transgender older adults who are racially underrepresented, immigrants, and other groups experiencing multiple forms of marginalization (Asti et al., 2024; Oswald et al., 2023; Velasco et al., 2023; Witten, 2014b). This article aims to address these gaps by examining health challenges and supports among transgender older adults—many of whom are racially marginalized and immigrants—through an Equitable Aging in Health framework (Perone et al., 2025c).

**Figure 1.**
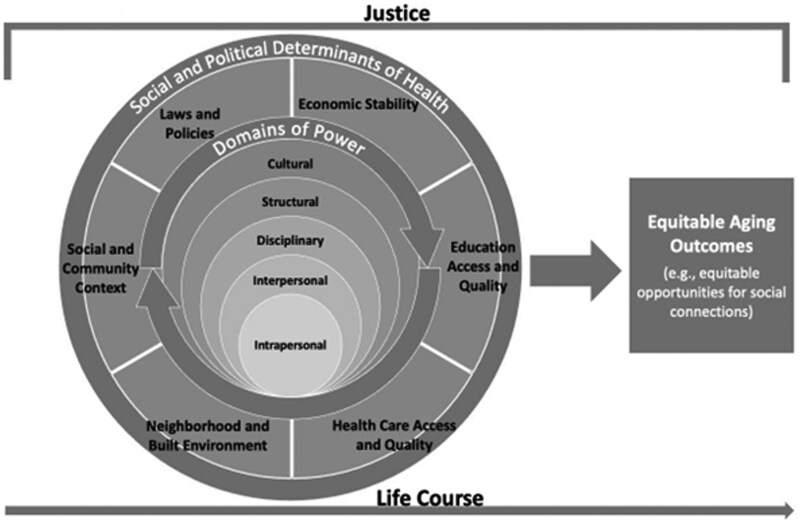
Equitable aging in health conceptual framework (Perone et al., 2025c) .

The Equitable Aging in Health conceptual framework uses a multi-level lens of power (intrapersonal, interpersonal, disciplinary, structural, cultural) to link social and political determinants of health in six categories (i.e., laws/policies, economic stability, education access/quality, health care access/quality, neighborhood and built environment, and social and community context) (Perone et al., 2025c) (see Figure 1).

This conceptual framework foregrounds power as the engine that drives social and political determinants of health and potential interventions for equitable health outcomes across the life course. Instead of looking at health access as a separate issue that affects health outcomes, for example, it presents a tool for conceptualizing the myriad ways in which disparities in social and political determinants of health can shape life experiences. For example, an older woman who experienced pay discrimination throughout her life may have less of an economic safety net after her husband dies and may struggle with paying her utility bills, mortgage or rent, and other costs associated with aging in place that increase her risk of experiencing homelessness for the first time after age 50—a growing issue in the United States (Perone et al., 2025a). This conceptual framework underscores the importance of multi-level, multi-systems interventions that acknowledge and address her grief, history of economic disparities, and housing precarity. Applying this conceptual framework to transgender aging, for example, helps illustrate how an individual interaction between a healthcare provider and transgender patient is connected to laws (at the systems/structural level) that ban gender-affirming care. Experiences in housing discrimination (from individual landlords and discriminatory housing policies) also shape health disparities across the life course (e.g., substandard housing and exposure to toxic mold can elevate breathing issues across the life course). Transgender older adults—especially transgender older adults experiencing multiple forms of marginalization—experience complex intersections of disparities across levels and systems that shape their experiences in health. Likewise, a long history of discrimination has necessitated creative forms of resistance and support (Fabbre, 2017; Fredriksen-Goldsen et al., 2024; Li et al., 2023; Perone, 2024) that also reflect interconnected levels and systems. This study aims to examine this complexity as it relates to transgender older adults through the following community-driven research questions: (1) How do intersecting systems (e.g., housing, economics, social services) and levels (e.g., intrapersonal, interpersonal, disciplinary, structural, cultural) shape health-related challenges for transgender older adults? and (2) How do intersecting systems and levels shape health-related supports for transgender older adults?

## Research design and methods

This community-engaged study incorporates data from 23 focus groups with 208 LGBTQIA+ older adults in California to examine challenges, thriving and surviving strategies, and recommendations regarding health, housing, social services, and caregiving. Data were collected from June through October 2024. Eighteen community partners collaborated in refining the focus group protocol, recruitment, and dissemination. Focus groups are particularly beneficial for exploring complex phenomena and needs among underrepresented communities (Perone et al., 2025b; Redden et al., 2021) and when participants have something in common and share a stake in the issue being discussed (Barbour & Morgan, 2017). Focus groups allow participants to bounce off ideas, diversify opinions, and flesh out issues more comprehensively (Prasad et al., 2022).

### Focus groups

Of the 23 focus groups, 20 were inter-categorical (included both transgender and cisgender older adults), and three were intra-categorical (included only transgender older adults) (McCall, 2005). Intra-categorical focus groups foreground the complexity of experience *within* a particular social position or intersection (McCall, 2005; Perone et al., 2025b; Tinner et al., 2023) to explore complexity among transgender older adults who were diverse in age, race, gender, and sexual orientation. Inter-categorical focus groups foreground the complexity of experience *between* transgender and LGB+ cisgender older adults (McCall, 2005; Tinner et al., 2023). Incorporating both types of focus groups allowed for rich conversation in which participants could both build off comments from other transgender older adults and distinguish their experiences from those of cisgender participants.

Focus groups lasted approximately 2 hr each. Three of the 23 focus groups were virtual to accommodate rural locations and mobility concerns of the participants. All three intra-categorical focus groups with transgender older adults were in-person. In-person focus groups took place at a location identified by community partners throughout various regions in California. Focus groups ranged in size from 4 to 13, with an average size of nine participants. The intra-categorical focus groups with transgender older adults ranged from 9 to 12 participants. Two were conducted in Spanish, and one was conducted in English. Participants self-selected their pseudonyms for the study. Each focus group included two trained facilitators. Participants received a $50 gift card. This study was approved by a university research institutional review board and conforms with the 1964 Declaration of Helsinki and its later amendments.

### Participants

Community partners were heavily involved in recruiting a purposive sample of LGBTQIA+ older adults who are racially underrepresented, transgender older immigrants, and other underrepresented LGBTQIA+ communities that tend to be less visible in research, funding, and services in the United States (Abreu et al., 2022; Hwahng et al, 2022; Pipkin & Clarke, 2024). This project included one lead community partner (a nonprofit serving LGBTQIA+ older adults in Northern California and overseeing a statewide LGBTQIA+ aging coalition) who helped identify additional community partners throughout the state. The research team’s deep expertise in LGBTQIA+ community-building, advocacy, and services helped identify additional community partners for this project. Eighteen community partners recruited participants through a variety of avenues, including word-of-mouth, social media, flyers, and group meetings. Community partners included organizations from 17 counties throughout California that served primarily LGBTQIA+ communities generally, LGBTQIA+ older adults, transgender adults, older immigrants, Black lesbians, Black adults, Indigenous adults, and older adults generally. Four community partners also specifically focused on services and supports around health issues (e.g., addiction, HIV/AIDS, cancer). Each partner received a stipend of $1,250 for assisting with one focus group or $1,500 for assisting with more than one focus group. While the community stipends helped offset some of the costs for outreach, staff time, meals provided, facility space, and other costs, these expenses exceeded the allotted stipend, and community partners were predominantly motivated by the need for this data and interest in building a community around this topic.

In the inter-categorical focus groups, eight participants identified as transgender. In the intra-categorical focus groups, 29 participants identified as transgender. The total sample for this project included 208 LGBTQIA+ older adults, of whom 37 (19%) identified as transgender. Nearly 80% of participants had annual incomes below $30,000. Transgender older adults ranged from 46 to 75 in age. The median age was 55. While most aging-related research begins at age 50, we incorporated transgender participants who were as young as 46, given research on accelerated aging for populations experiencing multiple forms of marginalization (Pereira & Banerjee, 2021; Valencia et al., 2023) that applies to our transgender adult participants. Our community partners also provided expertise about the experiences of transgender aging, especially low-income and racially underrepresented transgender adults (who experienced intersecting oppressions regarding gender identity, race, and class), which suggested lowering the age for inclusion. See Table 1 for specific details about their intersecting positionalities.

**Table 1.** Participant sample of transgender older adults (*N *= 37).

Characteristic	*N* participants^a^	%
**Age**		
** 45–50 **	7	18.92
** 51–60 **	17	45.95
** 61–74 **	5	13.51
** 75+**	2	5.41
** Over 50, but exact age unknown **	6	16.22
**Race/ethnicity **		
** Hispanic/Latine **	25	67.56
** White **	6	16.21
** Black **	4	10.81
** Native**	2	5.40
** Asian**	1	2.70
** Not answered **	1	2.70
**Sexual orientation**		
** Straight/heterosexual^b^**	20	54.05
** Gay or lesbian^c^**	4	10.81
** Bisexual^d^**	6	16.22
** Queer^e^**	7	18.92
** Pansexual^f^**	5	13.51
** Two-spirit^g^**	3	8.11
** Same-gender loving^h^**	2	5.41
** Demisexual^i^**	1	2.70
** Write-In (transgender, transtop, what feels right’) **	3	8.11
**Current Gender **		
** Woman/transgender woman**	28	75.66
** Man/transgender man**	7	18.92
** Genderqueer/nonbinary/two-spirit^j^**	7	18.92
**Immigrant status **		
** Immigrant **	23	62.16
** Undetermined^k^**	14	37.84
**Income**		
** $0–$30,000**	29	78.38
** $31,001–$59,999**	2	5.41
** $60,000–$89,999**	2	5.41
** $90,000+**	3	8.11
** Missing**	1	2.70

a Numbers do not total to 37 or 100% in several categories where participants checked multiple categories.

b Straight or heterosexual refers to someone primarily attracted to people of a sex or gender different from their own.

c Gay or lesbian refers to someone primarily attracted to people of the same sex or gender.

d Bisexual refers to someone attracted to more than one gender, though not necessarily simultaneously or to the same degree. This can sometimes be used synonymously with pansexual.

e Queer encompasses a range of identities and orientations and can include individuals who do not exclusively identify as straight or who identify as non-binary or gender-expansive. While some older adults dislike this term because it was historically used as a slur, some transgender older adults and younger LGBQ+ older adults identify with this term.

f Pansexual refers to someone who has the potential to be attracted to people of any gender, though not necessarily simultaneously or to the same degree. This can sometimes be used synonymously with bisexual.

g Two-spirit is more commonly used among some Indigenous and First Nations people to describe someone who is not cisgender and/or heterosexual.

h Same-gender loving is more commonly used among Black Americans as an alternative to gay or lesbian.

i Demisexual refers to someone who experiences some sexual attraction but only in certain situations (e.g., after forming a strong emotional or romantic connection).

j Genderqueer and nonbinary refer to someone who identifies outside the binary categories of man and woman.

k We did not ask a specific question about immigration status on our demographic survey but conducted two focus groups specifically with transgender older immigrants. For transgender participants outside these focus groups, we were unable to determine whether they were immigrants, unless they otherwise disclosed this in their focus group.

### Data analysis

We used the Equitable Aging in Health conceptual framework to guide the analysis, which highlights intersections among social and political determinants of health (i.e., laws and policies, economics, education, healthcare/access, neighborhood and built environment, social and community context) and multiple levels (i.e., micro, mezzo, macro) (Perone et al., 2025c). Because we employed a conceptual framework and community-driven goals (to understand challenges and supports) to guide our analysis, we adopted an iterative coding approach using directed content analysis (Ge et al., 2022; Shieu et al., 2023) in Dedoose (2023). A directed content approach uses proposed codes based on a conceptual framework, hypotheses, or literature (Hsieh & Shannon, 2005; Staniland et al., 2023), as well as community goals. We created an initial codebook using a priori themes from the framework, as well as line-by-line coding of the first five transcripts. We incorporated additional codes as we analyzed more data (Ge et al., 2022). Using a team model for qualitative research, including multiple coders, can enhance rigor by infusing and carefully considering diverse positionalities and reflexivity through the research process (Saunders et al., 2023).

The final codebook had twelve parent codes (i.e., caregiving, discrimination, health, housing, intimate partner violence, safety, etc.) and up to nine child codes under each parent code (e.g., health challenges, health supports, health suggestions). We developed research memos throughout the data collection and analysis process. We incorporated recognized practices for qualitative team coding for rigor and trustworthiness, including transparency through thick and rich descriptions (de Medeiros, 2024), developing detailed reference materials to guide the coding team (Giesen & Roeser, 2020), and establishing a strong and supportive management structure to gradually build training skills (Giesen & Roeser, 2020) and to compare codes and resolve disagreements through discussion (Terrizzi et al., 2023). Through this process, we organized concepts and examined connections among these concepts (Terrizzi et al., 2023).

To analyze and combine data from intra- and intercategorical focus groups, we matched pseudonyms with demographic data provided in participant surveys (which they completed immediately prior to the focus groups). Participants used the same pseudonyms for the survey and focus groups, and participants announced their pseudonyms every time they made a comment in a focus group (e.g., “This is Little Deer speaking”). We also separately identified in research memos specific comments that participants made referencing social locations (e.g., race, gender) outside the core intracategorical focus group. For example, if “Ebony,” an older Black transgender woman, participated in a focus group for older lesbians (the intracategorical focus group in which she participated), we also included her data when analyzing data from other focus groups, including transgender older adults generally (or Black lesbian older adults). This approach allowed us to incorporate a more nuanced intersectional design.

This research team was intentionally comprised of researchers who reflected at least one of the core positionalities in each focus group regarding race, ethnicity, gender identity, gender, sexual orientation, and language. This approach helped build trust and rapport with community partners and participants and contributed diverse perspectives during data collection, analysis, and dissemination. Most of the research team shared a common disciplinary perspective (social work); although there was wide variation in additional training and disciplinary background, including biology, gerontology, law, sociology, psychology, and public health. Some team members were also from geographic regions near or similar to where the focus group was conducted. Through weekly meetings and research memos, we regularly reflected on how our social positions, identities, epistemological and philosophical beliefs, lived experiences, and disciplinary/professional training shaped our inquiry throughout this project.

## Findings

The findings below are segmented into two categories based on community-driven research questions: challenges and supports. Participants discussed challenges related to healthcare access, housing, employment and economics, and violence. They also shared existing supports related to community connections, financial support, educational workshops, homesharing programs, and legal support.

### Challenges

When asked about their biggest challenges, transgender older adults identified healthcare access, housing, employment and economics, and violence. Transgender older immigrants also described how these challenges interacted with language, work, immigration status, including limited assistance due to their immigration status, economic instability exacerbated by language barriers, and workplace discrimination and aging—particularly for those in sex work—as well as the persistence of discrimination and hostility they sought to escape. Many of the participants also discussed how intersections of aging and disability shaped how they experienced these challenges.

#### Healthcare access

Transgender older adults described numerous experiences of discrimination that make accessing healthcare difficult—and sometimes dangerous. Many reported that hospitals fail to provide appropriate care and do not offer medical information relevant to their needs. Some struggle with chronic illnesses and newly diagnosed conditions but face difficulties in obtaining consistent, quality treatment. Disrespect and misgendering by medical providers were common issues. Some doctors refused to use a patient’s identified pronouns even when they were listed, and clinics continued to refer to individuals by incorrect names and pronouns despite legal name changes. The lack of gender-affirming care led to feelings of exclusion and mistreatment, with calls for healthcare staff to be better educated on transgender identities and fraud prevention—including educational interventions that recognize differences between someone using a name or gender that best reflects them versus someone trying to commit healthcare fraud. For example, Carmela, a transgender older Latina, reported both the importance of health access and the realities of discrimination:

The health system for trans women is very important. We sometimes face issues like STIs, COVID-19, or the need for critical surgeries. Where can we, as trans women, go for all these needs? I’m saying this because a friend told me that she went to a hospital using a female name, but because of her appearance, she was told, “No, you are a man, and we cannot treat you as a woman.”

Limited access to in-person medical appointments, particularly since the pandemic, further complicated participants’ ability to receive necessary care from providers they could trust and with whom they could build relationships that are sometimes easier to forge through in-person interactions. Bamby, a transgender older Latina and Indigenous immigrant, noted that “access to health care is not as good as it used to be. Before the pandemic, we had in-person appointments with doctors, but now, we only have appointments by phone, mostly by phone.” While this is a reality for many people seeking healthcare, relationships with trusted healthcare providers may be especially important for transgender patients (given rampant discrimination) that can be maintained and strengthened through in-person appointments.

In another focus group, Betty, an older Asian American transgender woman, raised concerns about health access: “In a lot of places, it’s much more difficult to access any sort of gender-affirming care, or the waitlists are like years long.” Betty also noted how healthcare access—specifically to gender affirming care—impacts experiences regarding safety and inclusion because older adults who are visibly transgender “can’t leave their house…without facing at least harassment and possibly violence.” Carmela, Bamby, and Betty’s comments underscore the complexity of wanting opportunities to build trust and relationships with providers in person while also needing to minimize exposure to violence and harassment when leaving one’s home to secure healthcare.

#### Housing

Transgender older adults faced a scarcity of affordable housing and were often only able to live in their current housing conditions as in-home caregivers. Some experienced predatory housing scams that required deposits for non-existent housing. Older transgender Latina immigrants described experiences of chronic homelessness, discrimination in housing programs, and inadequate government-assisted housing, with restrictive conditions and poor infrastructure, while limited support and bureaucratic barriers further hindered access to stable shelter. Serving as a caregiver to others also provided housing—until that care ended as described by Axel, a white genderqueer transgender adult:

I live with my mother, and that’s one of the benefits of being her caretaker, is I get to live in her place with her. However, when she passes away, I am most likely going to have to [move].

Housing instability also negatively impacted the mental (and physical) health of transgender older adults, as described by Kathy, an older transgender Latina immigrant below:

Now, I have a partner who is supporting me, but I have to prostitute myself to cover my expenses. I am a 59-year-old person, so prostitution is difficult for me. I’m not 20 or 30 anymore. Sometimes, men, clients, prefer young women. I have struggled a lot, and I have never had—I was in two shelters, but I left because I didn’t like the environment there. Being there makes me feel bad, so I don’t feel like staying there. Other times, thank God, I have someone to live with. I don’t have a safe place to stay because I don’t know if things will go wrong tomorrow, and I’ll have to run away and go back to the shelter.

While Kathy currently benefited from partner support, she described a precarious housing situation that left her feeling as if her housing was unsafe and unreliable. She also noted how prior shelters where she lived negatively impacted her mental health, which left her not wanting to stay there.

Sofia, an older transgender Latina immigrant, also described how substandard housing impacted her sense of well-being, including while she recovered after gender-affirming surgeries:

I’ve lived in the same building for 13 years. In that building, my apartment has sprung leaks at least 10 times. They keep moving me to different apartments, and the same thing keeps happening. Why? The building is too old, and the walls are too damp inside. After my apartment leaked four times, I said, “This isn’t right. I can’t live here.” I've been asking to be moved to a different apartment or housing unit, but what do they do instead? They tell me they can’t because of my immigration status. It’s happened too many times already. They have moved me to a different apartment whenever they needed to repair my original one. During my time in that apartment, the ones they’ve moved me to also had leaks. I just can’t seem to find any comfort. I’ve had to endure all of this during my transition, after my surgeries.

Bamby, a transgender older Latina and Indigenous immigrant, added that being homeless created structural conditions that led them to testing positive for HIV.

Things didn’t go well for me in my country…. I became homeless. I told my brothers that I was gay, and they kicked me out of the house. I spent some time on the streets alone. From there, I fell into drugs, alcoholism, and sex, and I tested positive for HIV and AIDS.

#### Employment and economics

Transgender older immigrants discussed how economic instability and job discrimination profoundly and negatively impacted their lives, as noted by Rey, an older transgender Latino immigrant:

I have gone to interviews. I can see how they’re looking at me. You can tell when people are looking at you. Their eyes say everything. They see you and think, “Okay, you’re a trans man, so you’re actually a woman.” And they don’t give me the job because of that. All those things affect our lives.

Susy, an older transgender Latina immigrant, added her own experiences of discrimination:

I am trying to leave behind what I was doing in Mexico to build a better life. I am looking for work. I’ve applied at McDonalds and many other places. I’ve made phone calls, and they tell me to come in person. However, when I show up and they see that I’m a trans woman, they tell me the position has been filled and that they’ll call me back.

Susy described how, despite her aspiration to earn money as a stylist, restrictions around licensing, as well as intersecting barriers regarding limited access to education, housing precarity, and immigration status, have also required her to engage in sex work to earn money:

In the city [where I’m from in Eastern Mexico], I worked as a stylist. I came here looking for a better life, trying to get off the streets. I have no education; I didn’t even finish elementary school. In my case, I’ve always wanted to get off the streets and quit sex work to have a better life. I came looking for a better life when I arrived [here]. However, what I’ve found is that there is no work. I want to work as a stylist, but I need a license. I came here illegally and with nothing. No one can hire me, so my only option is to walk the streets to make some money by selling my body.

Madonna, an older transgender Latina immigrant, added that while “we get a lot [of benefits], unfortunately, not all of us qualify.” Erika, another transgender Latina immigrant, described how the termination of an in-home caregiving position (which provided housing and stability) left her unhoused: “I took care of a man for 18 years, and when he died, they threw me out on the street.”

#### Violence and bias

Transgender immigrants reported safety concerns and violence, particularly in the wake of the pandemic. Many described feeling constantly in danger, experiencing verbal harassment and degradation simply for walking in public spaces. Some had been physically assaulted multiple times near their homes, reinforcing a sense of insecurity in their daily lives. Housing placements often put them in high-risk areas with prevalent drug and alcohol use, sex work, and vandalism, where they were exposed to further threats to their safety and personal dignity.

Despite hopes for a safer future, many transgender older immigrants described feeling dismayed by the discrimination they have experienced—even in more inclusive places in California, as described by Miroslava, an older transgender Latina immigrant:

We came here to escape the transphobia we faced in our countries. Back there, as soon as you started working, people would call you things like, “You faggot, bitch,” and so on. It’s the same here.

Betty, an older Asian American transgender woman, connected the importance of violence and being visibly transgender.

I have some friends, they really want to transition, but they can’t because they can’t leave their house looking anything other than their assigned gender at birth without facing at least harassment and possibly violence.

For Betty’s friend, concerns about violence and harassment stymied efforts to even begin the process of expressing their authentic self. In another focus group, Selena, an older white transgender woman, noted that while she continues to experience harassment, it has morphed into a new form of bias as she has aged: “I’m always the butt of everybody’s joke at work, and teased, and made fun of. It’s gotten better since I got older. People started calling me sir, and they fear me now sometimes. I don’t know why.” For her, being feared by others has provided some level of protection; although, her current experiences of harassment underscore potential dangers associated with being visibly transgender.

Violence and concerns for safety were also tied to housing, caregiving, and health, as reported by Débora, an older transgender Latina immigrant.

I am a transgender woman who is over 50 years of age and caring for a disabled person. I have found myself in a difficult situation where the building where I’ve lived for 30 years has been sold. It is difficult to find a safe place for a disabled person and also for me because I am a transgender woman, and a lot of violence is happening in the streets.… I have spoken to my husband’s social worker, who should help me find a place suitable for Miguel (my husband) because we cannot be where there are smokers, drinkers, or drug addicts because it is not good for his health. I haven’t received an answer.

Débora is not just battling concerns for safety; she is also navigating the intersection of violence with affordable housing that provides a safe and healthy place for her husband and her to live—as well as a social service system with a history of excluding and dismissing people of color.

### Strengths and supports

While transgender older adults described many challenges, they also reported strengths in their lives, including community support, gender-affirming healthcare providers, economic support, educational workshops, homesharing programs, and legal support.

#### Community support

Support from other transgender community members and nonprofits serving transgender older adults provided important safety nets, connections, and assistance. Vera, an older transgender Latina immigrant, noted the following:

Moving to San Francisco makes me feel safe. I’ve been here for approximately 14 months. As a trans girl, I feel welcome by the community. I feel the groups protect me more. I have done well. I’ve received help from different groups in the form of housing and vouchers to use at Target and such.

Participants also described how nonprofits and other transgender older adults helped them navigate access to healthcare, health providers, therapy, and insurance coverage. For example, Bamby reported improved healthcare, therapy, and medications after being diagnosed with HIV when she was able to access support through an HIV-related nonprofit.

Other participants emphasized the importance of having a sense of belonging and self-acceptance that often came from families of choice/friends and community spaces that offered inclusive environments, including spiritual exploration where being one’s authentic self was welcomed. For example, Rufus, a white transgender older adult, connected the importance of community in combating loneliness: “I just want to say I am happy to have found community here…I’m not alone.” Little Deer, an Indigenous, genderqueer two-spirit older adult, also underscored the importance of community for people in recovery:

A lot of us have trauma in our lives…. The only other support that I know of is within the recovery community that I belong to. I’ve been to lots of open meetings that were for genderqueer gay people that are open. They were the coolest meetings, you know what I mean? There’s support and of course the people that are in that community with me, the people I sponsor, the people that are my support and stuff like that.

Little Deer’s comments also emphasize the importance of having targeted community supports for transgender older adults that are culturally responsive for transgender older adults who are racially minoritized, including immigrants, and in recovery from substance or alcohol use.

#### Gender-affirming healthcare providers

Many participants underscored the importance of gender-affirming healthcare providers and resources from nonprofits and friends about who provided gender-affirming care and how they could access it, especially with limited economic means. For example, Bamby, a transgender older Latina and Indigenous immigrant, reported that after she learned she was HIV-positive, she “thought [she] was going to die soon.” She added that she “had counselors, therapists, psychologist, doctors, and people who had my back, who helped me and guided me. They supported me a lot. I received therapy and medicine.”

Location also played an important role for many participants who talked about disparities in accessing care based on where they lived, as illustrated below by Betty, an older Asian American transgender woman:

A lot of places it’s much more difficult to access any sort of gender-affirming care or the waitlists are like years long. We’re really lucky to be here. It would be nice if the rest of the world would catch up.

Betty’s comment underscores both an appreciation for being able to access gender-affirming care where she currently lives and a recognition of her own privilege in accessing this care based on her location. It also highlights the complexity that many transgender older adults described of feeling grateful for supports they had but also a desire to elevate awareness about existing gaps for others. Even though she herself had gender-affirming services, her intentional mention of continued gaps in care also underscores a strength in how transgender older adults invoked community and education (even when their current needs were met in one area) to advocate for the needs of transgender older adults that they may not personally know but who may share a common historical experience of exclusion and community.

#### Economic support

Several participants discussed the importance of financial support from nonprofit organizations, including those providing support for various chronic conditions and disabilities. Madonna, an older transgender Latina immigrant, noted the following:

Where I live, they help me pay rent, and that’s a big help. My rent is only about $1,200, but because I am in bad health, I qualify for many kinds of support. There is an [AIDS support] organization, and they help me by giving me $500. My rent comes down to just $800 with that help.

Housing support and rent subsidy programs stemming from AIDS and HIV-support organizations underscore the intersections of health and housing for many transgender older adults.

#### Educational workshops

Transgender older adults underscored the importance of educational workshops, particularly those that provided information to support their mental health, as illustrated by comments from Lucera, a transgender older Latina immigrant.

Education has helped me greatly in many ways. The different workshops I took through organizations have helped me strengthen my mental health and progress professionally. I am very grateful for the workshops the organizations offer constantly.

In referencing educational and social supports for substance use, Jessy, a transgender older Latina immigrant, noted that programs in Spanish are helpful because “not many substance abuse counselors speak Spanish.” Fanny and Madonna also noted that educational workshops were helpful when delivered in Spanish and noted obstacles they experienced when Spanish translation was only provided remotely. For example, they described how they were invited to an educational meeting organized by and for Latinas and Latinos. But it was delivered in English, and they “understood only half of what was being said.” The organization subsequently called an interpreter who translated remotely by video. While Fanny and Madonna appreciated the eventually translated information, they ultimately described feeling unwelcome and praised other organizations and programs for transgender Latinas and Latinos that provide information in Spanish for them. Fanny and Madonna’s experiences underscore the complexity of how educational workshops can provide important strengths but also sources of challenges when linguistic support is not provided.

#### Shared housing and homesharing

Several transgender older adults discussed the benefits provided by shared housing or nonprofit homesharing programs. Shared housing involves unrelated people sharing a house or apartment with common spaces, and nonprofit homeshare programs are led by high-touch nonprofit organizations that facilitate and support matches and formal shared living agreements between two unrelated people who live together and mutually benefit from a shared exchange of rent and/or services (e.g., grocery shopping, laundry) (Perone et al., 2025a). For example, Little Deer, an Indigenous, genderqueer two-spirit older adult, described how transgender and genderqueer communities build community and support by living together:

There are ways that communities can come together, even the trans, and in the genderqueer communities, living together and being able to afford these rents and stuff in a communal way. There’s that advantage because the rents are high everywhere.

In response to a question about current supports, Rufus, a white transgender older adult, described the benefits of homesharing as a source of support:

The [nonprofit] has a program where you can live with a senior. They will do the matching. They will do the vetting of both sides, and then they’ll meet with you together, and everything is agreed ahead of time … I’ve seen people exchange low rent for help with groceries or whatever. I think that’s a great program.

In response to Rufus’s comments about this homesharing program, Little Deer added that Rufus was describing a “home match program” and both Little Deer and Betty, an older Asian American transgender woman, underscored how homesharing programs can help minimize challenges of finding affordable housing due to high costs of rent, poor credit, and housing scams.

#### Legal support

Several participants noted the importance of legal supports, including education on how to report discrimination by health care providers. For example, Olga, a transgender Latina immigrant, shared information with the group about calling the Medical Board of California when experiencing discrimination by nurses and doctors.

Regarding discrimination in hospitals, clinics, rental apartments, etc, that’s why there are laws, and by those laws, those professionals receive their credentials. Hence, you can call the Medical Board in Sacramento. The Medical Board of California in Sacramento is here for when a doctor or a nurse discriminates against you. You can call and report them so that the medical supervisors can take action against that doctor. Under the law, if you ask a doctor for his ID number or license number so that you can report him to the Medical Board of California in Sacramento, an order for abuse is immediately issued.

Fanny, a transgender Latine immigrant, added that information shared in Spanish can be especially helpful for transgender older immigrants for understanding and exercising their legal rights. Several participants also noted that providing legal support in Spanish can help build trust among immigrant communities, which is especially important for transgender immigrants who described legal challenges related to discrimination across multiple systems because they are an immigrant, transgender, aging, and predominantly speak Spanish.

## Discussion and implications

While implied in many responses, some participants explicitly connected challenges in housing, employment, and economics to their health, whose connections can become more visible through the Equitable Aging in Health conceptual framework. Participants discussed how they experienced violence associated with being visibly transgender that was tied to limited access to gender-affirming care. Many transgender older adults struggled to secure affordable housing, shaped by discrimination in the neighborhood and built environment that elevated exposure to predatory housing scams and apartments with toxic mold and flooding. Serving as an in-home caregiver provides both supports and risks for housing precarity and underscores the need for better supports for transgender older adults providing in-home care (and caregivers generally). Several transgender older adults, especially Latina immigrants, discussed intersections of housing, employment, and safety that often required seeking housing in shelters. Discrimination in shelters, however, prompted some to resort to living on the streets, an experience that transgender people in other studies have reported (Jackson et al., 2022; Mottet & Ohle, 2006). Employment discrimination provided limited economic opportunities that left many participants in precarious housing tied to in-home caregiving or sex work. And, homelessness limited economic opportunities and led some transgender older adults to engage in sex work to earn money that exposed them to STIs and other health conditions, which aligns with other research (Coleman et al., 2022; Feinstein, 2019; Stutterheim et al., 2021). Trans-related discrimination further limited economic and employment opportunities that became cumulative across the life course. Limited economic security and unstable housing based on accumulated inequities across the life course created limited options for where to live, and many transgender older adults described living in constant danger. Frequent safety concerns can create a constant sense of panic and stress that can produce allostatic overload that wears the body down, elevating risks for co-morbidity and mortality (Caceres, 2017; Rich et al., 2025).

Participants also discussed how various health conditions and disabilities created additional challenges that intersected with aging as they tried to find affordable, safe, and stable housing, employment, and culturally responsive healthcare. All these systems were inextricably interconnected in ways that shaped their health and well-being. Moreover, these interconnected challenges persisted across levels. Individual experiences of discrimination in housing were tied to cultural biases about trans people that may have elevated risks of experiencing more discrimination (e.g., predatory housing scams) because of limited economic means and housing options. Organizational practices and policies may have negatively shaped health among transgender older immigrants through eligibility tied to immigration status or programs only offered in English. While this study cannot make causal claims, given the qualitative research design, the depth and breadth of this data underscore just how these interconnected systems and levels shaped health-related challenges for transgender older adults.

The strengths and supports reported by transgender older adults in this study also reflect the intersection of various systems and levels. Families of choice, including other transgender older adults, shared essential networks of gender-affirming providers and strategies for resisting structural barriers that negatively impacted their health and well-being, which aligns with other research on transgender older adults (Fabbre & Gaveras, 2020; Li et al., 2023; Toze et al., 2023; White Hughto & Reisner, 2018 ). Community nonprofits at the organizational level and neighborhood and built environment provided financial assistance, spaces for social connection, access to gender-affirming care, and programs for safe and affordable housing. Educational workshops delivered by individuals through nonprofit organizations, agencies, or community groups provided another important source of information about how to navigate a complex labyrinth of structures that were often very different from the countries of origin of some of our participants. Workshops also provided culturally responsive mental health support. Workshops delivered in Spanish were especially helpful for many of the older transgender Latina and Latino immigrants in this study. Policies and organizational structures that provided avenues for reporting discrimination also provided symbolic and sometimes actual support that several participants found helpful.

Data further underscore how challenges and supports were not siloed experiences. For example, being an informal (or formal in-home) caregiver to other older adults provided housing and economic security that could be abruptly terminated when that care recipient died—thus providing both supports and challenges. But when caregiving ended, they were left without supports of their own. Experiences of caregiving (and precarious housing) shaped perceptions of physical and mental health and well-being among several transgender older participants. Likewise, educational workshops provided critical information (support) but less so when only delivered in English (challenge) for many of the transgender older Latina and Latino immigrants in this study.

While intersecting systems and levels shaped health-related challenges and supports for all transgender older adults in this study, intersecting positionalities (e.g., race, income, immigration status) further shaped their experiences, which aligns with recent research on transgender older adults who are racially minoritized (Walker et al., 2023). Positionalities reflect one’s positions of power in relation to others in various social, political, and economic structures, cultural contexts, and interpersonal dynamics (Perone et al., 2025b). The sample of transgender older adults in this study predominantly comprised older adults experiencing multiple forms of marginalization related to economic hardship, racism, disability, sexism, and older age that also presented diverse strategies for surviving and thriving. However, older transgender Latina and Latino immigrants reported challenges and supports that were connected more deeply to basic needs. For example, being undocumented (or living in the United States on a visa) and/or limited economic opportunities and supports created reliance on certain arrangements (e.g., in-home caregiving) that left some transgender older adults more vulnerable to sex work, homelessness, and negative health outcomes like STIs. They also, however, underscored the importance of support networks from other transgender immigrants who could help buffer some of these challenges by providing informal networks of support, knowledge about gender-affirming providers, and sometimes temporary housing. Transgender older adults with disabilities reported challenges accessing services and securing safe and stable housing, too, which was mitigated through community support and knowledge sharing about culturally responsive and affirming accessible services. While transgender older adults living with HIV often had access to financial support for housing through federal housing programs, some of our participants were ineligible for those programs because of their immigration status. These varying circumstances required different types of supports. Transgender older adults excluded from formal support systems (e.g., federal financial support for housing, health insurance for gender affirming care) relied more heavily on informal supports from families of choice or community networks, or went without those supports.

Participants’ sources of strengths and support further highlight how policymakers, practitioners, and researchers could develop services, programs, and policies across systems and levels that could better address the complexity of the challenges they encountered. For example, a nonprofit focused on providing gender-affirming physical and mental health care for transgender adults could seek funding for an innovative program that also incorporates peer support for housing (e.g., homesharing/tiny homes), builds community, and supports aging-in-place and in community, specifically an affirming community. Transgender older adults in the United States are already invoking nontraditional models to support community-based affordable housing for and by transgender communities (Perone et al., 2025d), and a gender-affirming clinic could leverage this existing practice or collaborate with other community-based organizations to develop a health-based intervention that supports not only their gender-affirming care but the housing they need to feel safe to seek care and recover after receiving care, as well as maintain positive health and well-being.

This study has several limitations. First, this study collected data only from LGBTQIA+ older adults in California in the United States. Additional studies outside California and the United States are needed. Moreover, fewer participants identified as transgender men, and more research is needed on older transgender men, including Latinos, and other men who are racially and ethnically minoritized. With a more diverse sample (including one that includes transgender Latino immigrant men), research could also compare challenges and supports to create interventions that are also attentive to gender. Relatedly, intersectional research is inherently challenging, and it can be difficult to disentangle challenges and strengths stemming specifically from gender identity, race, language, age, or immigration status, as these experiences are often deeply interconnected. However, qualitative research using an intersectional framework (e.g., inter/intra-categorical design) can help unpack some of these nuances. Furthermore, the data were collected right before a significant administrative transition in the United States, which eliminated funding for data collection on LGBTQIA+ communities (Mueller, 2025) and ushered in new policies that target gender affirming care and the rights of transgender people (Exec. Order No. 14168, 2025; Exec. Order No. 14183, 2025). This shift continues to increase vulnerabilities (and possibly new creative survival strategies) among many transgender older adults and underscores the importance of this research. Follow-up data could provide comparisons between these social and political moments and further unpack surviving and thriving strategies amidst these policy shifts.

Overall, this study offers several important contributions and innovations. First, it provides much-needed data from the voices of transgender older adults, including racially minoritized older adults and immigrants, who are often excluded from aging and health-related services, programs, and policies. “Minoritized” refers to people from groups who have experienced historical and systematic marginalization and is focused less on the quantity of people but more on power and equity (National Academies of Sciences, Engineering, and Medicine, 2023). While not generalizable, these data provide important context, understanding, and nuance about transgender aging that would be difficult to surface through quantitative methods, given the depth of the data and complexities of intersecting positionalities. The findings also illuminate complexities among intersecting systems that necessitate practice and policy solutions that transcend traditional service and agency boundaries. For example, programs solely focused on providing housing vouchers may be insufficient if not also paired with interventions that address economic barriers and discrimination that perpetuates the need for housing vouchers. Nonprofits and service providers thus could point to this study when seeking funding support for innovative programs that invoke multiple systems and levels of support. Researchers could build on this study’s data to conduct similar studies of transgender older adults outside of California that would increase our understanding of challenges and strengths, particularly among transgender older adults who are low-income, racially minoritized, or immigrants. Policymakers and agency leaders could point to data from this study to support the need for a more nuanced multi-systems level approach to develop prevention and intervention programs that address health disparities among transgender older adults, as well as older adults more generally. Moreover, this study applies an innovative conceptual framework to help understand how systems and levels are connected and shape health experiences for transgender older adults. It moves beyond identifying potential interventions at one level or system and presents a framework and empirical data that policymakers and practitioners can build from to provide culturally responsive and more holistic approaches to serve transgender older adults. Ultimately, this study underscores the importance of policies, practices, and programs for transgender older adults that attend to the complex intersections of the systems and levels that shape diverse health experiences of transgender older adults and how experiences in these systems may reflect challenges, supports, or both, given different contexts.

## Data Availability

Due to IRB and ethical restrictions with some of the individual community partners and participants, we are currently unable to share the raw qualitative data. However, examples of the coding process and focus group protocol are available upon request. This study was not preregistered.
